# Single-walled carbon nanotubes functionalized with sodium hyaluronate enhance bone mineralization

**DOI:** 10.1590/1414-431X20154888

**Published:** 2015-12-04

**Authors:** M.A. Sá, H.J. Ribeiro, T.M. Valverde, B.R. Sousa, P.A. Martins-Júnior, R.M. Mendes, L.O. Ladeira, R.R. Resende, G.T. Kitten, A.J. Ferreira

**Affiliations:** 1 Departamento de Morfologia, Universidade Federal de Minas Gerais, Belo Horizonte, MG, Brasil; 2 Departamento de Física, Universidade Federal de Minas Gerais, Belo Horizonte, MG, Brasil; 3 Departamento de Bioquímica e Imunologia Universidade Federal de Minas Gerais, Belo Horizonte, MG, Brasil

**Keywords:** Osteoblast, Nanotechnology, Carbon nanotubes, Sodium hyaluronate, Tissue engineering, Osseointegration

## Abstract

The aim of this study was to evaluate the effects of sodium hyaluronate (HY), single-walled carbon nanotubes (SWCNTs) and HY-functionalized SWCNTs (HY-SWCNTs) on the behavior of primary osteoblasts, as well as to investigate the deposition of inorganic crystals on titanium surfaces coated with these biocomposites. Primary osteoblasts were obtained from the calvarial bones of male newborn Wistar rats (5 rats for each cell extraction). We assessed cell viability using the 3-(4,5-dimethylthiazol-2-yl)-2,5-diphenyl-2H-tetrazolium bromide assay and by double-staining with propidium iodide and Hoechst. We also assessed the formation of mineralized bone nodules by von Kossa staining, the mRNA expression of bone repair proteins, and the deposition of inorganic crystals on titanium surfaces coated with HY, SWCNTs, or HY-SWCNTs. The results showed that treatment with these biocomposites did not alter the viability of primary osteoblasts. Furthermore, deposition of mineralized bone nodules was significantly increased by cells treated with HY and HY-SWCNTs. This can be partly explained by an increase in the mRNA expression of type I and III collagen, osteocalcin, and bone morphogenetic proteins 2 and 4. Additionally, the titanium surface treated with HY-SWCNTs showed a significant increase in the deposition of inorganic crystals. Thus, our data indicate that HY, SWCNTs, and HY-SWCNTs are potentially useful for the development of new strategies for bone tissue engineering.

## Introduction

The advent of nanotechnology has enabled researchers to develop new functional materials, devices, and systems, with promising potential in the healthcare and medicine fields (1,2). In this context, carbon nanotubes (CNTs) have emerged as one of the most exciting candidates for use in bone tissue engineering because of their remarkable mechanical, thermal, and electrical properties, and their ability to functionalize with other polymers (3-7).

Recent studies have shown that CNTs, alone or combined with other biomaterials, promote osteoblast cell proliferation and bone tissue deposition (7-11). CNTs act as a scaffold, allowing osteoblast cells to adhere, spread, and proliferate (12-15). In addition, CNTs may control events such as crystal nucleation and growth of inorganic bone components (16).

Sodium hyaluronate (HY)-functionalized CNTs (HY-CNTs) can accelerate bone repair and regeneration of tooth sockets in healthy rats (17) and can restore the pattern of trabecular formation in diabetic rats (18). HY is a glycosaminoglycan that is found in the extracellular matrix of mammalian tissues (19), which also stimulates osteoprogenitor cells to migrate, proliferate, and differentiate into osteoblasts (20,21) by binding to cell surface receptors, such as CD44 and the receptor for hyaluronic acid-mediated motility (20,22). Thus, it appears that HY and HY-CNTs can actively induce bone formation by activating osteoblasts. In this regard, HY-CNTs can be used to coat titanium implants, which by themselfs are not capable of inducing bone formation (23). Several studies have shown that titanium coated with CNTs or hyaluronic acid induces better cell adhesion and proliferation, along with increased bone formation around dental implants (23-25).

Although there have been studies examining the synthesis, characterization, and properties of CNTs, further *in vitro* and *in vivo* studies are needed to better comprehend the effects of this nanomaterial on bone repair and regeneration before it can be safely used in humans (26). In this study, we aimed to evaluate the effects of HY, CNTs, and HY-CNTs on the biological behavior of primary osteoblasts, and to investigate the deposition of inorganic crystals on titanium surfaces coated with these biocomposites.

## Material and Methods

### Primary osteoblast cell culture

Primary osteoblast cells were obtained from calvarial bones of newborn (2-4 day old) Wistar rats. Experimental protocols were performed in accordance with the institutional guidelines approved by the Ethics Committee in Animal Experimentation of the Universidade Federal de Minas Gerais, Brazil (protocol #217/2009) and the National Institutes of Health Guide for the Care and Use of Laboratory Animals. After euthanasia, calvariae were dissected, washed with Hank’s balanced salt solution (HBSS), treated with 100 µg/mL gentamicin (Gibco, USA), and then washed with a solution composed of HBSS, α-minimum essential medium (α-MEM) (Gibco), and 190 µg/mL gentamicin. During washing, calvariae were cleaned with a sterile lint-free wipe to remove blood and soft tissue, and then cut in two pieces. Thereafter, calvariae were sequentially digested with 1 mg/mL collagenase type II (Gibco) diluted in 0.25% trypsin for 5, 15, and 25 min at 37°C. The supernatant of the first digest was discarded and the cells were resuspended in α-MEM supplemented with 10% fetal bovine serum (FBS; Gibco), 100 µg/mL gentamicin, 5 µg/mL ascorbic acid (Sigma-Aldrich, USA), and 2.16 mg/mL β-glycerophosphate (Sigma-Aldrich). Samples were then pooled and filtered through 70 µm nylon filters (BD Biosciences, USA). Subsequently, the cells were counted and plated onto 24-well culture plates at a density of 2.5×10^4^ cells/well (27,28). The culture medium was changed every 2 days. When the cells reached 70-80% confluence, different concentrations of HY, single-walled CNTs (SWCNTs), and HY-functionalized SWCNTs (HY-SWCNTs) were added to the medium. The synthesis, purification, and characterization of SWCNTs and HY-SWCNTs was previously described by our research group (17,18).

### MTT cell viability assay

The 3-(4,5-dimethylthiazol-2-yl)-2,5-diphenyl-2H-tetrazolium bromide (MTT) cell viability assay (Sigma-Aldrich) is based on the ability of NAD(P)H-dependent cellular oxidoreductase enzymes in viable cells to convert the soluble substrate MTT into insoluble formazan crystals. The resulting purple formazan can be solubilized and quantified by spectrophotometric means and is proportional to the number of viable cells. For each treatment (HY, SWCNTs, and HY-SWCNTs), five concentrations (10 pg/mL, 1 ng/mL, 100 ng/mL, 10 µg/mL, and 1 mg/mL) were tested. After 48 h incubation, osteoblasts were subjected to a quick wash with HBSS, following which the MTT substrate was added (500 µg/mL) diluted in α-MEM, and the cells were maintained for 4 h in a CO_2_ incubator at 37°C. After the incubation period, a further wash was performed with HBSS. Then, a solution of isopropanol/HCl was added and the samples were agitated to promote the elution of the formazan crystals. The supernatant was quantified by measuring the absorbance values at 595 nm in a SpectraMax 250 microplate reader (Molecular Devices, Minneapolis, MN, USA). Two independent experiments were performed in duplicate.

### Quantification of apoptotic/necrotic cells

After incubation with the biocomposites (HY, SWCNTs, and HY-SWCNTs) for 48 h at a concentration of 100 ng/mL, primary osteoblasts cultured on coverslips were double-stained without fixation, as previously described (29). Hoechst 33342 (Molecular Probes, USA) and propidium iodide (PI; Sigma-Aldrich) were added to the culture medium at final concentrations of 1 µg/mL and 250 ng/mL, respectively, and the cells were incubated for 15 min at 37°C in the dark. Thereafter, the medium was removed and the cells were quickly washed with HBSS. The coverslips with adherent cells were removed from the 24-well culture plates and mounted on slides. The positive control was cells that were incubated with 50 µg/mL digitonin (Amend Drug and Chemicals Co., USA) for 5 min, as previously described (30). All experiments were done in triplicate. Five images of each well were captured and averaged to obtain a single value per region of interest (ROI). Images were acquired on a fluorescence microscope (Axio Imager M2 imaging system; Carl Zeiss, Germany) with a 20×0.5 objective, using Axiovision 4.8 software (Carl Zeiss) to create images. Exposure time was set with the same values for all data collected. For each ROI, images were separately acquired for Hoechst and PI staining, using specific filters sets compatible with the excitation-emission profiles of each fluorescent dye. Images were then analyzed using Image-J software (http://rsbweb.nih.gov/ij/). The background (auto-fluorescence) was subtracted and double-staining with Hoechst and PI was demarcated using a colocalization Image-J plug-in (31). For cell quantification, cells were defined as particles with a minimum diameter of 13 µm^2^. The number of double-labeled osteoblasts was normalized to the total number of cells stained by Hoechst.

### Mineralized bone nodule analysis by von Kossa staining

Primary osteoblasts were grown in the presence of HY, SWCNTs, or HY-SWCNTs (100 ng/mL) for 7 days. After this period, the medium was removed, and the cells were washed with HBSS and fixed with 70% ethanol for 24 h. Cells were then washed under running water for 10 min, followed by the addition of 1 mL 5% silver nitrate to each well and exposure of the plate to ultraviolet light for 1 h. Next, the silver nitrate was removed and the cells were washed thoroughly with distilled water. After washing, 1 mL of 5% sodium thiosulfate was added to each well for 5 min to remove unreacted silver (32). After staining, the plate was observed on an inverted microscope and photographed to capture the area of mineralization, which was indicated by black staining. Ten images per well were randomly obtained and the mineralized area was quantified using Image-Pro Plus software (Media Cybernetics, USA).

### Real-time polymerase chain reaction (RT-PCR)

The mRNA expression of genes related to bone repair was assessed by quantitative RT-PCR. Primary osteoblasts were grown in the presence of HY, SWCNTs, or HY-SWCNTs (100 ng/mL) for 3 or 5 days. Cells were trypsinized with 0.25% trypsin/ethylenediaminetetraacetic acid (Gibco) and washed with HBSS. Total RNA (n=4 wells per group) was isolated using Trizol Reagent (Invitrogen, USA), according to the manufacturer’s recommendations. The PCR amplifications were performed using the StepOnePlus Real-Time PCR System (Thermo, USA). Each 15-µL reaction contained 1 µL of cDNA, 1X Maxima SYBR Green/ROX qPCR Master Mix (Thermo), and 800 nM of each primer (Table 1). The PCR protocol comprised 5 min of denaturation at 95°C, 45 cycles of 1 min each for annealing and elongation at 60°C, and 10 s of denaturation at 95°C. Fluorescence was detected at the end of each extension phase. To exclude contamination of nonspecific PCR products, such as primer dimers, the dissociation curve analysis was applied to all products at the end of the cycle. Relative quantification of the expression of the target genes was performed using the comparative CT method, as previously described (33). Fold-changes in gene expression of the target genes are equivalent to 2^-ΔΔCT^.

**Table:**
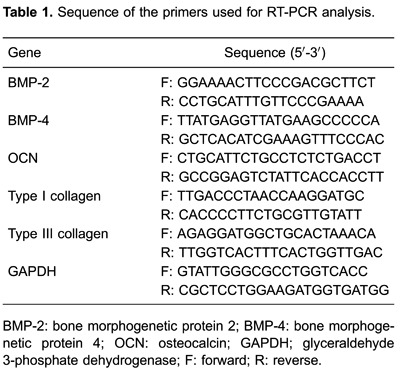

### Deposition of inorganic crystals on titanium surfaces

Titanium disks, approximately 4.6 mm in diameter and 1.9 mm in height, were prepared and 50 µL of HY, SWCNT, or HY-SWCNT gels was pipetted onto the disks. After the gels were spread evenly across the disk, the thickness of the gel was measured using calibrated digital calipers (Mitutoyo, Brazil). The gel layer measured approximately 1.5 mm. Subsequently, disks coated with the gels were placed in a drying oven at 40°C overnight. After drying, the disks were immersed in an undersaturated aqueous solution containing 0.5 mM CaCl_2_ and 0.25 mM Na_2_HPO_4_ (16). Silicon substrates were used as the negative control because they do not induce ion deposition and precipitation when immersed in undersaturated saline solutions. Uncoated titanium disks were also used as a control. Each disk was immersed in individual capped containers. The disks were maintained under slow stirring at room temperature for 28 days. After this period, they were washed with pure water to remove excess salt, dried at 37°C, and stored for morphological analysis by scanning electron microscopy. Additionally, energy-dispersive X-ray spectroscopy analysis was performed to identify chemical elements present in the sample.

### Statistical analysis

Data are reported as means±SE. Statistical analyses were performed using one-way ANOVA followed by the Newman-Keuls post-hoc test, or two-way ANOVA followed by the Bonferroni post-hoc test, as indicated in each figure legend (GraphPad Prism 5 software, USA). P<0.05 was considered to be statistically significant.

## Results

### MTT cell viability assay

**Figure 1:**
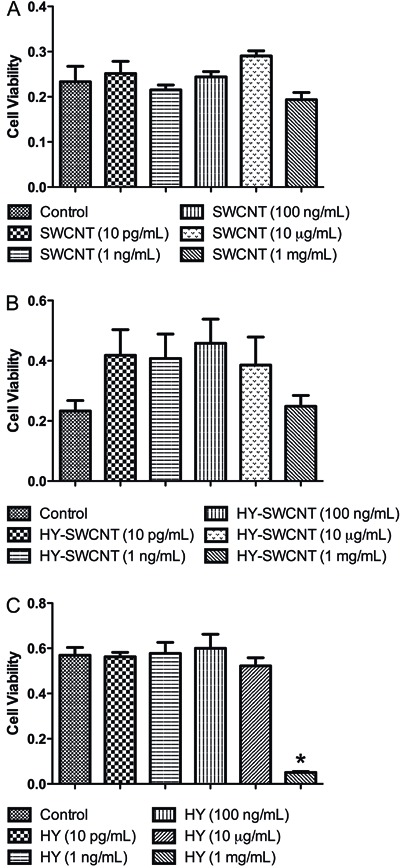
Cell viability evaluated by 3-(4,5-dimethylthiazol-2-yl)-2,5-diphenyl-2H-tetrazolium bromide assay. Cells were treated with single-walled carbon nanotubes (SWCNT; *A*), HY-functionalized SWCNT (HY-SWCNT *B*), or sodium hyaluronate (HY; *C*). A high concentration of HY (1 mg/mL) significantly reduced the viability of primary osteoblasts. *P<0.05 compared to control and other concentrations of HY (one-way ANOVA followed by Newman-Keuls post-hoc test).

No significant differences in viability were observed between the cells treated with different concentrations of SWCNTs or HY-SWCNTs compared with the control group (Figure 1A and B). Low concentrations of HY (10 pg/mL, 1 ng/mL, 100 ng/mL, and 10 µg/mL) also had no effect on cell viability. However, a high concentration of HY (1 mg/mL) significantly reduced the viability of primary osteoblasts after 48 h of treatment (Figure 1C).

### Quantification of apoptotic/necrotic cells

**Figure 2:**
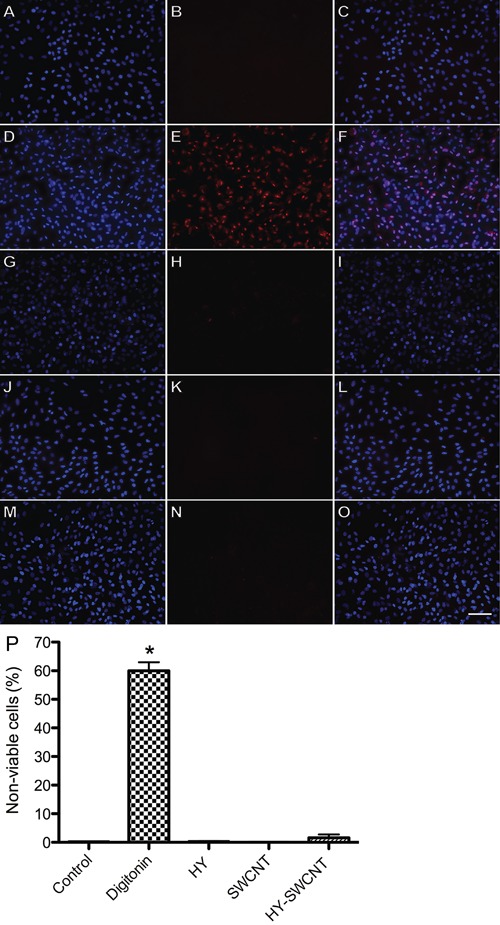
Double-staining with Hoechst and propidium iodide (PI). The images in the left column show the total number of cells stained with Hoechst, the middle column shows the cells with positive staining for PI, and the right column shows the merged images. Control cells (*A*-*C*) exhibited no double-staining. Positive control group cells (incubated with 50 µg/mL digitonin; *D*-*F*) showed a large number of double-labeled cells, indicating that the method was effective. Treatments with sodium hyaluronate (HY; *G*-*I)*, single-walled carbon nanotubes (SWCNT; *J*-*L*), and HY-functionalized SWCNT (HY-SWCNT; *M*-*O*) did not affect cell viability, which was confirmed by morphometric analysis (*P*). *P<0.05 compared to control, HY, HY-SWCNT, and SWCNT (one-way ANOVA followed by Newman-Keuls post-hoc test). Scale bar = 100 µm.

Cell viability was also assessed by immunofluorescence. The negative control group (untreated cells) contained viable cells (Figure 2A-C), while the group treated with digitonin exhibited dead cells (Figure 2D-F). The cells treated with HY (Figure 2G-I), SWCNTs (Figure 2J-L), and HY-SWCNTs (Figure 2M-O) showed little co-localization (PI and Hoechst), indicating that the viability of the osteoblasts was not affected by any of the biocomposites.

Morphometric analysis confirmed these results, with the groups treated with HY (0.3±0.1%), SWCNTs (0±0%), and HY-SWCNTs (1.6±1.2%) showing a low number of non-viable cells compared with the control group (0.2±0.1%). There were no significant differences among the treatment groups (Figure 2P). Together, these results confirm those obtained by the MTT assay.

### Measurement of mineralized bone nodules by von Kossa staining

**Figure 3:**
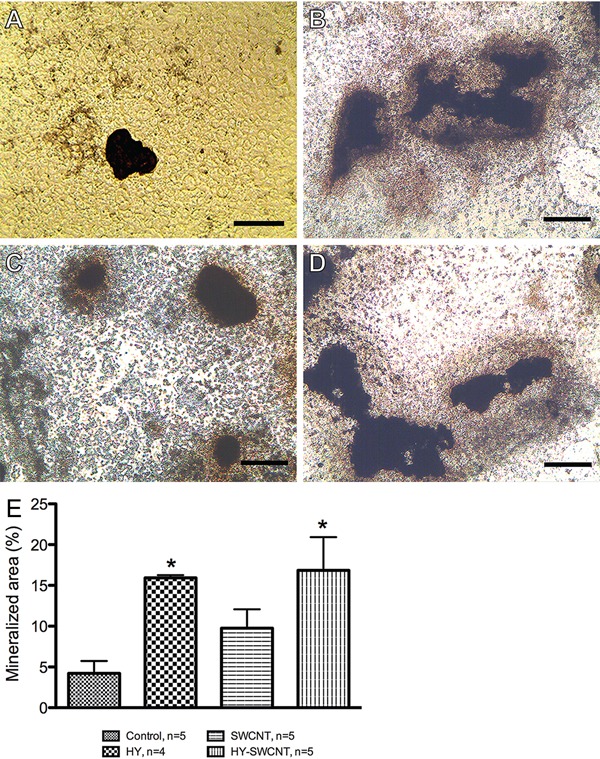
Von Kossa staining for the analysis of mineralized bone nodule formation. Photomicrographs show that treatment with sodium hyaluronate (HY; *B*), single-walled carbon nanotubes (SWCNT; *C*), and HY-functionalized SWCNT (HY-SWCNT; *D*) increased the mineralization area compared with the control (*A*). Morphometric analysis (*E*) showed that treatment with HY and HY-SWCNT significantly increased the formation of mineralized bone nodules. *P<0.05 compared to control (one-way ANOVA followed by Newman-Keuls post-hoc test). Scale bar = 100 µm.

Histological imaging of cells treated with HY (Figure 3B), SWCNTs (Figure 3C), and HY-SWCNTs (Figure 3D) revealed that these biomaterials increased the mineralized bone nodule area compared with the control group (Figure 3A). The largest and most organized nodules were observed in wells containing HY or HY-SWCNTs (Figure 3B and D). In agreement with these data, the morphometric analysis revealed that treatment with HY and HY-SWCNTs significantly increased the area of mineralized bone nodules (control: 4.2±1.5%; HY: 15.9±0.3%; HY-SWCNTs: 16.8±4.1%) (Figure 3E).

### RT-PCR

**Figure 4:**
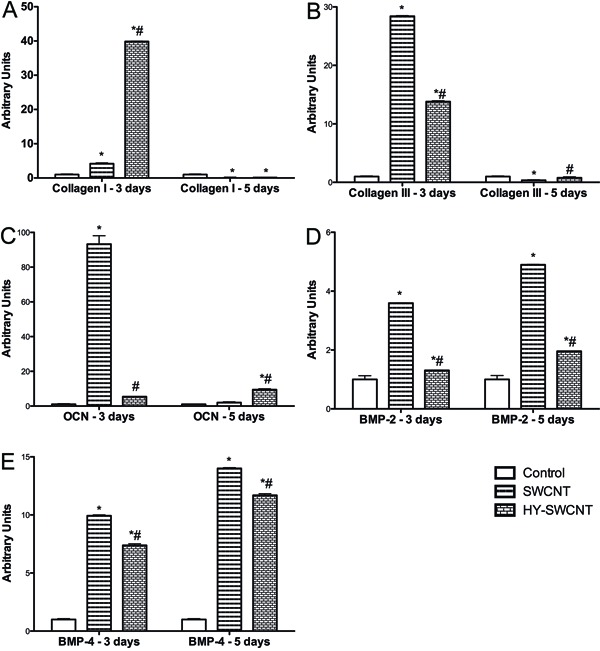
mRNA expression of bone repair factors. Type I collagen (*A*), type III collagen (*B*), osteocalcin (OCN) (*C*), bone morphogenetic protein-2 (BMP-2) (*D*), and BMP-4 (*E*) after treatment with single-walled carbon nanotubes (SWCNT) or sodium hyaluronate (HY)-SWCNT. *P<0.05 compared to control and ^#^P<0.05 compared to SWCNT (two-way ANOVA followed by Bonferroni post-hoc test).

HY-SWCNTs and SWCNTs significantly increased the expression of type I collagen after 3 days of treatment, with HY-SWCNTs exhibiting a higher effect compared with SWCNTs. In contrast, after 5 days of treatment, the expression of type I collagen was lower in both of these groups (Figure 4A). Similarly, the expression of type III collagen was increased after 3 days of treatment with HY-SWCNTs or SWCNTs, with a higher effect exhibited by SWCNTs. After 5 days of treatment, the expression of type III collagen was lower only in the group treated with SWCNTs (Figure 4B). Osteocalcin (OCN) expression was increased only in cells treated with SWCNTs after 3 days of treatment. Furthermore, after 5 days of treatment, the expression of OCN was increased only in cells treated with HY-SWCNTs (Figure 4C). The expression of bone morphogenetic protein (BMP)-2 (Figure 4D) and BMP-4 (Figure 4E) was significantly increased after 3 and 5 days of treatment with HY-SWCNTs or SWCNTs, with a higher effect exhibited by SWCNTs.

**Figure 5:**
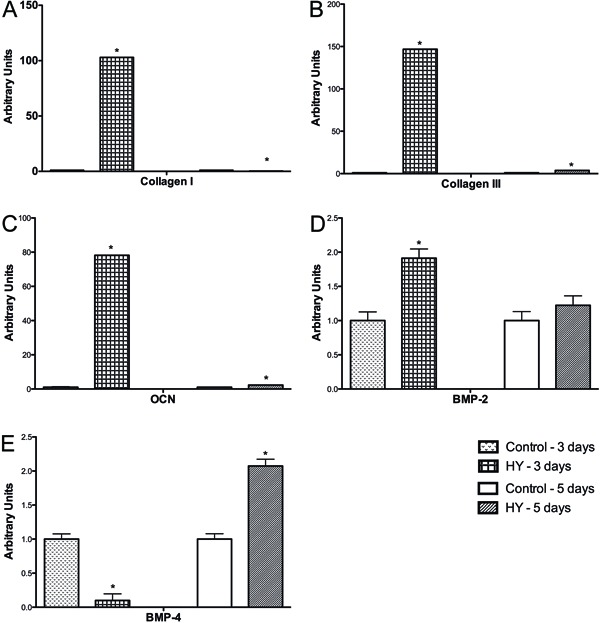
mRNA expression of bone repair factors. Type I collagen (*A*), type III collagen (*B*), osteocalcin (OCN) (*C*), bone morphogenetic protein-2 (BMP-2) (*D*), and BMP-4 (*E*) after treatment with sodium hyaluronate (HY). *P<0.05 compared to control (two-way ANOVA followed by Bonferroni post-hoc test).

Following treatment with HY, type I collagen expression was increased after 3 days and subsequently decreased after 5 days of treatment (Figure 5A). The expression of type III collagen and OCN was significantly increased in cells treated with HY after 3 and 5 days treatment compared with the control group (Figure 5B and C). Moreover, the expression of BMP-2 was increased only after 3 days of treatment (Figure 5D), whereas BMP-4 expression was decreased after 3 days and increased after 5 days of treatment in cells incubated with HY (Figure 5E).

### Induction of deposition of inorganic crystals on titanium surfaces

**Figure 6:**
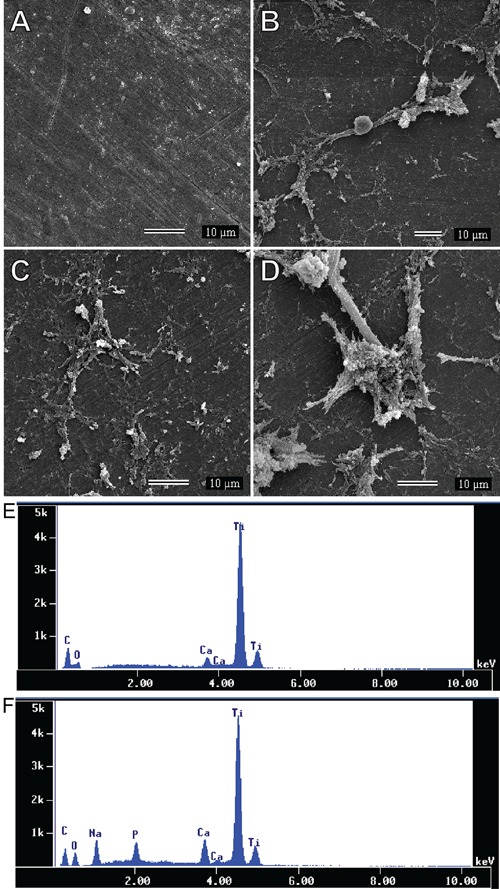
Scanning electron microscopy and energy dispersive X-ray spectroscopy analyses of titanium disks. Scanning electron microscopy revealed that untreated titanium disks (*A*) exhibited few and small particle deposits. Titanium disks coated with sodium hyaluronate (HY; *B*), single-walled carbon nanotubes (SWCNT; *C*), and HY-functionalized SWCNT (HY-SWCNT; *D*) showed higher particle deposition compared with uncoated disks. Energy dispersive X-ray spectroscopy analysis showed that the deposited particles in untreated titanium disks contained only calcium ions (*E*), while in disks treated with SWCNT, HY or HY-SWCNT (*F*), the particles contained calcium, sodium, and phosphorus ions.

After 28 days of immersion in an undersaturated aqueous solution of CaCl_2_ and Na_2_HPO_4_, untreated titanium disks exhibited only minor deposition of particles on their surfaces (Figure 6A). Alternatively, disks treated with HY (Figure 6B), SWCNTs (Figure 6C), and HY-SWCNTs (Figure 6D) showed higher deposition of particles compared with untreated titanium disks. Notably, the highest and most organized deposition of particles was seen on disks treated with HY-SWCNTs. Silicon substrates did not exhibit any deposition of particles after 28 days of immersion (data not shown).

Energy-dispersive X-ray spectroscopy analysis demonstrated that the deposited particles on untreated titanium disks were composed only of calcium ions (Figure 6E), while particles present on disks treated with HY, SWCNTs, and HY-SWCNTs were formed of calcium, sodium, and phosphorus ions (Figure 6F). The peaks of titanium were attributed to the composition of the disks and the carbon ions incorporated during the preparation of the samples, whereas the peaks of oxygen were due to the oxidation process.

## Discussion

The main findings of the present study were that HY, SWCNTs, and HY-SWCNTs at low concentrations did not exhibit cytotoxic effects on primary osteoblasts. Moreover, these biocomposites increased the deposition of inorganic crystals in osteoblast cultures and on titanium surfaces. Because CNTs can affect absorbance reading in the MTT assay, it is not a reliable technique for measurement of cell viability in their presence (34-36). Thus, other techniques, or even a combination of MTT with other assays, would be better suited to evaluate the viability of cells incubated with CNTs (8,9,11,15). In view of this fact, we used a second method to analyze cell viability by double-staining with Hoechst and PI. Again, we found that SWCNTs and HY-SWCNTs did not compromise cell viability. Accordingly, Pan et al. (37) demonstrated that 0.5% multi-walled CNTs increased the proliferation of rat bone-marrow-derived stromal cells (BMSCs) and did not cause cell death when evaluated by MTT and live/dead assays. In addition, HY did not interfere with the viability of primary osteoblasts, even at high concentrations (38).

HY and CNTs have previously been shown to augment the deposition of mineralized bone nodules by increased expressions of osteopontin, OCN, and runt-related transcription factor 2 (RUNX2) (8,11,38). In addition, we showed that HY and HY-SWCNTs enhanced the deposition of mineralized bone nodules, likely because of the ability of these biocomposites to increase mRNA expression of important proteins for bone formation, such as type I and III collagen, OCN, BMP-2, and BMP-4. Surprisingly, despite the upregulation of these important proteins for bone formation, SWCNTs did not increase the deposition of mineralized bone nodules, likely because of the absence of HY in its formulation.

Our results show that HY, SWCNTs, and HY-SWCNTs induce higher deposition of inorganic crystals on titanium disks composed of calcium, sodium, and phosphorus ions. Other studies have shown that CNTs induce nucleation and growth of hydroxyapatite crystals under physiological concentrations of calcium and phosphate (10,16). Moreover, it has been demonstrated that the interaction of CNTs with polymers or other nanomaterials enhances their ability to induce mineralization (24,39) and osteogenesis (40). Indeed, we found that the highest and most organized deposition of particles occurred on disks treated with HY-SWCNTs, probably because of the ability of HY to accelerate the bone repair process (20,21). However, one limitation of our data is that no measurements of uniformity and thickness of the layer of HY, SWCNTs, or HY-SWCNTs on the surface of the disks were performed after the drying procedure, which might influence the deposition of inorganic crystals.

In summary, we demonstrated that HY, SWCNTs, and HY-SWCNTs induce bone mineralization by acting in both organic and inorganic components of this tissue. Thus, these biocomposites are potentially useful for developing new bone tissue engineering strategies and improving the osseointegration of titanium.
